# Participation of the ATR/CHK1 pathway in replicative stress targeted therapy of high-grade ovarian cancer

**DOI:** 10.1186/s13045-020-00874-6

**Published:** 2020-04-21

**Authors:** Patrycja Gralewska, Arkadiusz Gajek, Agnieszka Marczak, Aneta Rogalska

**Affiliations:** Department of Medical Biophysics, Faculty of Biology and Environmental Protection, Institute of Biophysics, University of Lodz, Pomorska 141/143, 90-236, Lodz, Poland

**Keywords:** ATR kinase, CHK1, ovarian cancer, PARP, replication stress, targeted therapy

## Abstract

Ovarian cancer is one of the most lethal gynecologic malignancies reported throughout the world. The initial, standard-of-care, adjuvant chemotherapy in epithelial ovarian cancer is usually a platinum drug, such as cisplatin or carboplatin, combined with a taxane. However, despite surgical removal of the tumor and initial high response rates to first-line chemotherapy, around 80% of women will develop cancer recurrence. Effective strategies, including chemotherapy and new research models, are necessary to improve the prognosis. The replication stress response (RSR) is characteristic of the development of tumors, including ovarian cancer. Hence, RSR pathway and DNA repair proteins have emerged as a new area for anticancer drug development. Although clinical trials have shown poly (ADP-ribose) polymerase inhibitors (PARPi) response rates of around 40% in women who carry a mutation in the BRCA1/2 genes, PARPi is responsible for tumor suppression, but not for complete tumor regression. Recent reports suggest that cells with impaired homologous recombination (HR) activities due to mutations in TP53 gene or specific DNA repair proteins are specifically sensitive to ataxia telangiectasia and Rad3-related protein (ATR) inhibitors. Replication stress activates DNA repair checkpoint proteins (ATR, CHK1), which prevent further DNA damage. This review describes the use of DNA repair checkpoint inhibitors as single agents and strategies combining these inhibitors with DNA-damaging compounds for ovarian cancer therapy, as well as the new platforms used for optimizing ovarian cancer therapy.

## Introduction

Ovarian cancer is considered to be one of the most lethal gynaecologic malignancies worldwide. It is the seventh most common cancer and the fifth leading cause of cancer-related deaths [1]. As a result of the absence of formal screening and the continued lack of early detection methods, the majority (around 80%) of patients are diagnosed at an advanced stage (III/IV) [2]. The 5-year survival rate of patients with high-grade serous ovarian carcinomas (HGSOCs) still ranges between 35 and 40% [3]. In 2019 in the USA, an estimated 22,530 women were diagnosed with ovarian cancer and 13,980 died from the disease [4].

Ovarian tumors can be divided into two types: type I ovarian cancers are composed of mucinous, endometrioid and low-grade serous carcinomas, while type II tend to grow more aggressively and include carcinosarcomas, undifferentiated carcinomas and high-grade serous carcinomas [5]. Moreover, almost all of the type II carcinomas, i.e. 96%, have TP53 mutation [6] and around half of HGSOCs carry an alteration in homologous recombination (HR) pathway genes, most commonly in breast cancer gene (BRCA) 1/2 [7]. Women carrying mutations in these genes have a lifetime risk of developing ovarian cancer of 36 to 60% for BRCA1 and 16 to 27% for BRCA2 [8].

The initial, standard-of-care, adjuvant chemotherapy in epithelial ovarian cancer (EOC) is usually a platinum drug, such as cisplatin or carboplatin, combined with a taxane, usually paclitaxel [9]. Cisplatin interferes with the DNA repair mechanism by crosslinking the purine bases of the DNA, and thus inducing apoptosis of cancer cells [10]. The standard regimen for advanced ovarian cancer has been expanded with bevacizumab, a recombinant humanized monoclonal antibody directed against vascular endothelial growth factor (VEGF) [11]. Other promising angiogenesis inhibitors are sorafenib and sunitinib [12, 13]. Since the addition of bevacizumab to the combination of standard chemotherapeutics, many other targeted anticancer agents have been studied in the hope of increasing the effectiveness of ovarian cancer treatment. Ovarian cancer cells often acquire resistance to common chemotherapy drugs such as cisplatin. If a tumor recurs within 6 months of cisplatin treatment, it is considered to be platinum-resistant [14, 15].

The aim of this article is to review the current knowledge of the targeting of DNA repair pathways in ovarian cancer. This review describes the use of DNA repair checkpoint inhibitors, especially poly (ADP-ribose) polymerase inhibitors (PARPi), ataxia telangiectasia and Rad3-related protein inhibitors (ATRi) and checkpoint kinase 1 inhibitors (CHK1i), as monotherapy/single agents, and their role in the treatment of patients with BRCA^mut^ ovarian cancer. It also briefly characterizes the rationale of therapies combining these inhibitors, as well as recent updates/advances in those therapies in vitro and in clinical trials.

## Replication stress and cell cycle disturbances in ovarian cancer

Increased understanding of the tumor repair pathways has revealed their significance in the sensitivity of cells to chemotherapeutic agents. DNA damage signalling pathways have a central role in detecting DNA damage and regulating its repair. Regulation of cellular responses to interference in these pathways by numerous extrinsic and intrinsic genotoxic agents leads to genomic instability and thus to cell death [16]. Replication stress is defined as perturbations in cell replication. In defence against disorders in the course of DNA biosynthesis, cells have developed a network of biochemical reactions that can be described as a response to replicative stress. Under conditions of replicative stress, the rate of DNA biosynthesis is decreased and the possibility of entering into mitosis is blocked until the expression of specific genes and activation of repair factors occurs. Ataxia telangiectasia mutated (ATM) and ataxia telangiectasia and RAD3-related (ATR) proteins share some phosphorylation targets, but their precise role in the intra-S phase checkpoint pathway may differ depending on the nature of stress involved [17]. The action of ATR/ATM kinases induces cascade signal transmission to effector proteins (e.g. checkpoint kinase 1/2 (CHK1/CHK2)). Both biochemical pathways function according to the following event patterns: DNA breaks–ATM–CHK2 and DNA breaks–ATR–replication block–CHK1.

In each of these pathways, the target substrate is CDC25 phosphatase. ATR also activates Dbf4-dependent kinases (DDK). The other checkpoint protein, WEE1 kinase, also keeps cyclin-dependent kinases (CDK) muted. Inactivation of CDK/DDK is pivotal for the inhibition of origin firings under replication stress [18]. WEE1, which belongs to the large Ser/Thr family of protein kinases, is known as one of the most essential molecules in executing cell cycle arrest at the G2/M checkpoint, which is pivotal for premitotic DNA repair [19]. WEE1 kinase coordinates the initiation of mitosis by antagonistic regulation of Cdk1/Cdk2. In a comparable manner to ATR and CHK1, it operates during regular undisturbed cell division and is involved in the preservation of genome integrity [20].

**Fig. 1:**
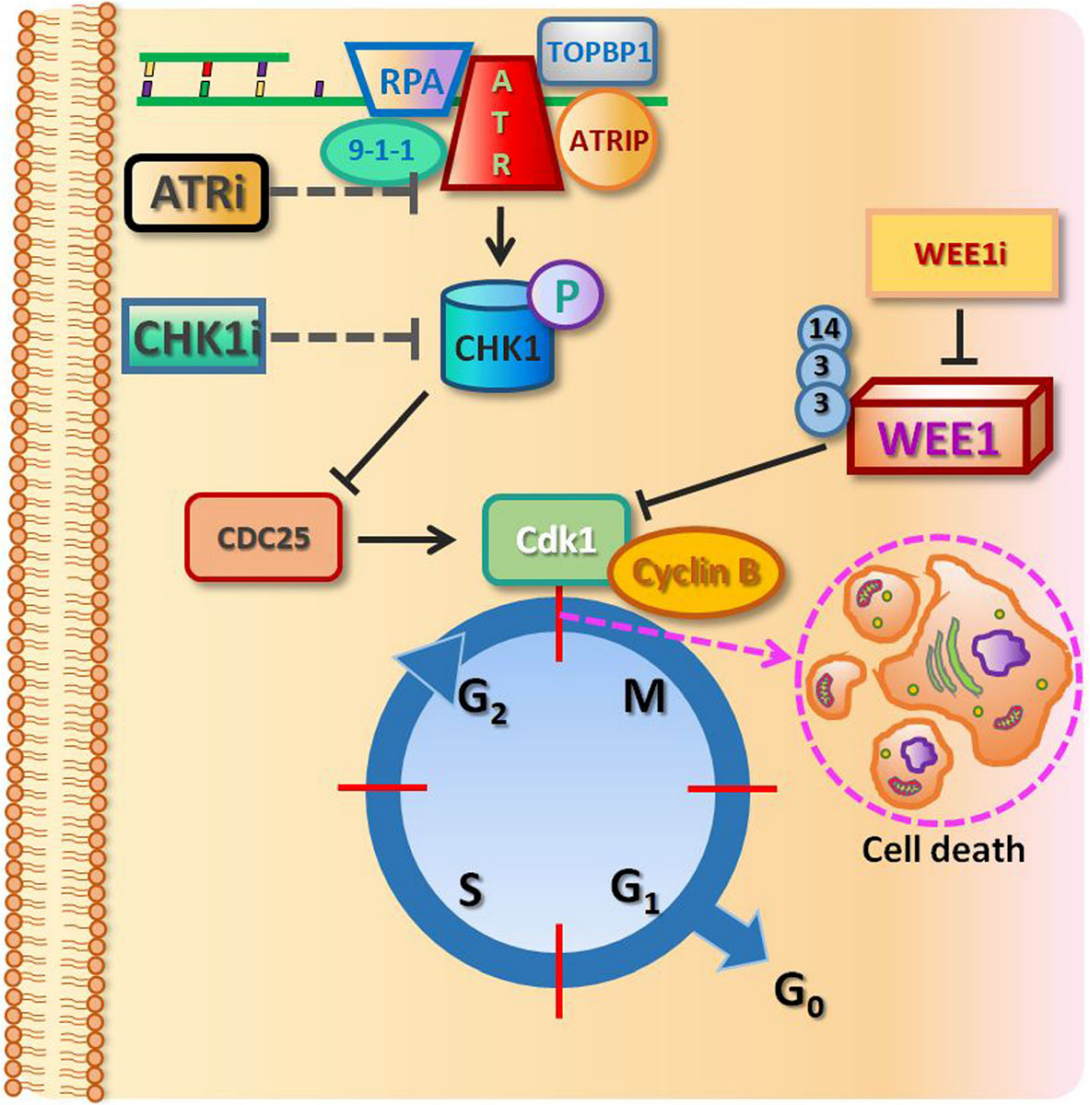
Participation of WEE1 kinases (Cdk1 inhibitors) and CDC25 phosphatases (Cdk1 activators) in the regulation of the activity of Cdk1 kinase during G2 and M phases. The binding of WEE1 kinases to 14-3-3 protein, which activates WEE1 kinases, may be carried out in two different ways—via the phosphorylation of ser642 (with participation of CHK1) or autophosphorylation. WEE1 inhibitor abrogates the G2/M checkpoint, resulting in cancer cell death

Cyclin A-Cdk1 and cyclin B-Cdk1 complexes play a major role in the regulation of mitosis. They are inactive until the late G2 phase, due to two separate processes: inactivation of Cdk1 kinase and inhibition of its activators—Cdc25A, Cdc25B and Cdc25C phosphatases. The WEE1 kinase family—nuclear (WEE1) and membrane (Myt1)—are responsible for inhibiting Cdk1 activity. WEE1 and Myt1 start to work when the cell goes from mitosis to the G1 phase of the new cycle. At this time, reduction of the new cyclin B-Cdk1 complex is required for cyclin B degradation [21]. In addition, their activity may be increased in the case of DNA damage and expression of CHK1 and CHK2 kinases, which phosphorylate a specific WEE1 serine (Ser642) and induce attachment of 14-3-3 protein [22]. Autophosphorylation of WEE1 proteins is also possible, which contributes to their positive regulation (Fig. 1). To allow mitosis to occur, WEE1 is phosphorylated by Polo-like Kinase 1 (PLK1), which triggers WEE1 degradation [23].

Cancer cells, due to mutations in the p53/pRb pathway, frequently exhibit a deficient G1-arrest and mostly depend on G2-arrest [24]. WEE1 inhibitors appear to have potential high efficiency in p53-deficient ovarian cancers, generating a condition of synthetic lethality among p53 mutant tumors. Inhibition of WEE1 has demonstrated its effectiveness in DNA damage as a consequence of unregulated replication and strengthened the effectiveness of DNA-damaging agents [25]. Until now, only one (the most potent and selective) WEE1 inhibitor (Adavosertib, also under the names AZD1775 and MK1775) was found to be useful in clinical trials (in both phases I and II). WEE1i is able to stimulate origin firing, with dynamics of action comparable to ATR and CHK1 inhibitors. However, the mechanism of its action is not fully understood and requires a more detailed investigation [26]. MK1775 increased the cytotoxicity of numerous DNA-damaging drugs (such as antimetabolites, topoisomerase inhibitors and DNA crosslinking agents) towards various cancer cells. WEE1i was highly effective in p53-deficient cells and cells with defects in DNA damage repair pathways [27]. MK1775 was also combined with carboplatin treatment and paclitaxel in platinum-sensitive recurrent ovarian cancer [25]. The first report to present clinical evidence of MK1775 enhancing the efficacy of carboplatin in TP53-mutated tumors was published by Leijen et al. in 2016, NCT01164995 [28]. It was shown that it was active in a wide variety of human tumor xenografts, including models of ovarian cancer, with limited single-agent clinical activity [29].

ATR participates in the response to single-stranded (ssDNA) and double-stranded DNA breaks (DSBs) and to a variety of DNA lesions that interfere with replication [30]. ATR promotes cell cycle arrest and repair of DNA or induces apoptosis if the repair systems are overwhelmed. As a consequence, CHK1 is activated. Proteins that are part of the DNA damage response (DDR) pathway are phosphorylated, including histone H2AX, breast cancer type 1/2 susceptibility protein (BRCA1/2), RAD51 and p53. In addition, inactivation of p53 leads to loss of activity of the G1 checkpoint, which favours G1–S transition [31]. Previous studies have shown that chemotherapeutics such as cladribine induce ATR-dependent phosphorylation of H2AX, a biomarker for DNA double-strand breaks, and the p53 suppressor protein [32, 33]. In response to DNA damage, ATR phosphorylates CHK1 protein, which in turn mediates CDC25A-C phosphorylation, leading to the blocking of CDK1 and CDK2 (thus preventing cell cycle progression). CHK1 can stabilize the replisome, possibly by targeting replication proteins (e.g. CDC6, minichromosome maintenance proteins 2–7 (MCM2-7)), and after resolving the replication problems can restart stalled replication forks. Functional changeability of the ATM/ATR–CHK2/CHK1–CDC25/CDK axis underlies the molecular foundation of the intra-S-phase checkpoint [34].

**Fig. 2:**
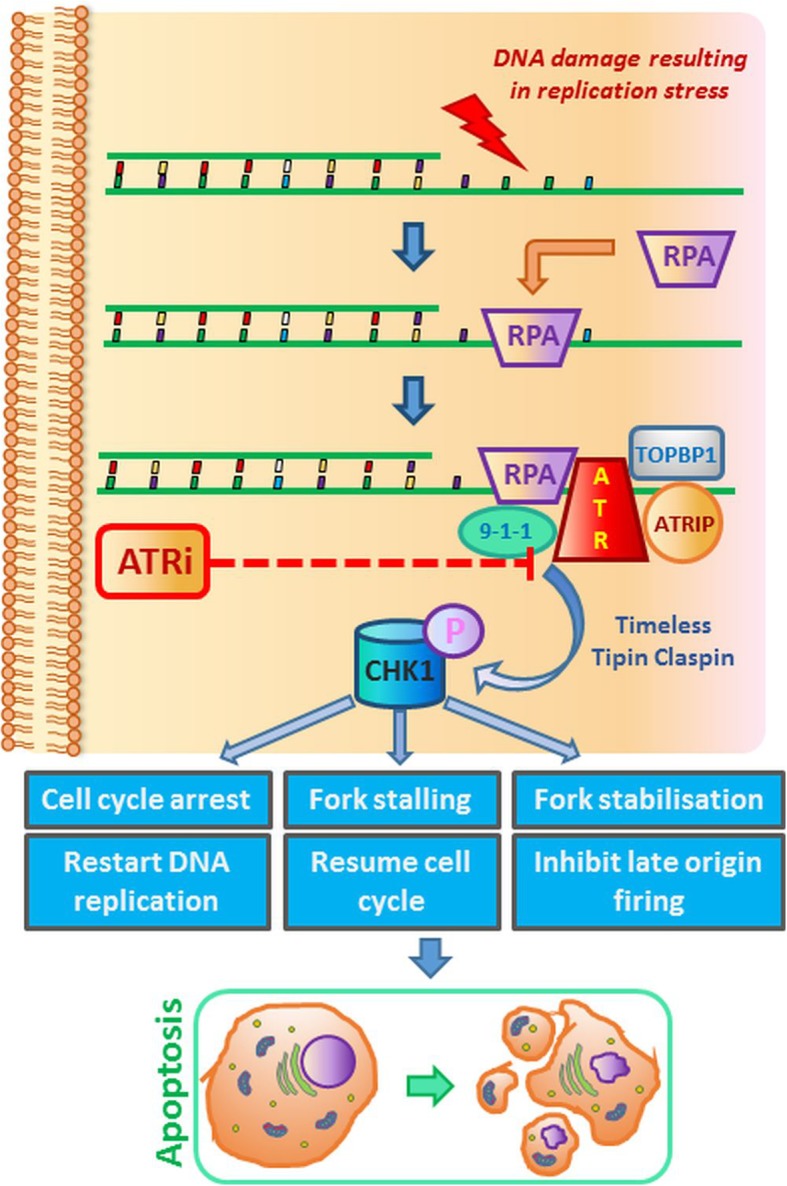
DNA damage and replication checkpoints. Anticancer drugs induce replication disorders. Replication stress is the effect of the slowing or stalling of replication fork progression. DNA synthesis inhibition or damage induces checkpoint responses controlled by the ATR–CHK1 pathway. DNA lesions delay entry to S-phase (G1 checkpoint), slow the replication of damaged DNA or prevent entry to mitosis (G2 checkpoint). Given that both PARP and checkpoint proteins prevent fork collapse, their corresponding inhibitors may increase the level of replication stress, genome instability and, in consequence, cell death

In response to replication stress, replication protein A (RPA) is the first to be loaded onto the unstable single stranded DNA (ssDNA), and the long stretches of RPA-coated ssDNA adjacent to the double-stranded DNA (dsDNA) act as a platform to trigger the ATR/CHK1 [35]. ATR combines with ATR-interacting protein (ATRIP), and their complexes are located in the cell nucleus at the sites of DNA damage [36]. The RAD9–RAD1–hus1 (9-1-1 complex) is required for the recruitment of DNA topoisomerase 2-binding protein 1 (TopBP1) [37]. The activators of ATR–ATRIP complexes are the TopBP1 and two other factors: replication factor C (RFC) and proliferating cell nuclear antigen (PCNA). During replication, RFC recognizes the sites of primer junctions of RNA with template DNA and assembles around them a toroidal protein homotrimer, PCNA, commonly named as sliding clamp, which determines the movement of DNA polymerases associated with it [38]. The first stage of the signaling pathway is the placement of cell cycle checkpoint protein Rad17 and ATR–ATRIP complexes in the damaged sections; the second is Rad17-dependent assembly of PCNA-type complexes around the DNA. The PCNA-type complexes support the activation of ATR molecules and, consequently, the phosphorylation of its substrates located within the chromatin, such as Rad17 and Rad9 [39]. When DNA errors and damage which are caused during the genetic material replication are not removed in time, the stalled replication forks are susceptible to fork collapse, leading to highly lethal DSBs (Fig. 2).

The disappearance of the function of the internal phase S control point, caused for example by the mutation of ATR, means that cells with damaged genetic material are blocked and are unable to enter mitosis despite the occurrence of non-replicated sections. Induction of DNA damage and activation of cellular responses, i.e. cascades of DNA damage signalling pathways, by a number of anticancer drugs result in cell cycle arrest at the G1/S and G2/M phases. When the amount of DNA damage is too high to allow the cell to survive, it is most often redirected to apoptosis [40]. Hence, DNA repair has emerged as a new area for anticancer drug development [41]. Numerous drugs are currently being tested in clinical trials; some of them are presented in Table 1. Most HGSOCs have mutations in TP53 and other genetic alterations associated with increased replication stress. Inhibitors of ATR and CHK1 are therefore promising drugs for ovarian cancer treatment.

**Table 1:** Clinical trials with chemotherapeutic agents causing replication stress used in ovarian cancer treatment. [clinicaltrials.gov] ^*^not applicable

Target	Name of drug/drugs	Objective of trial	Clinical trial phase	Clinical trial identifier
**PARP**	BMN 673 (talazoparib)	Patients with deleterious BRCA 1/2 mutation-associated ovarian cancer who have had prior PARP inhibitor treatment	2	NCT02326844
**PARP**	BSI-201 (iniparib) Carboplatin/ gemcitabine	Patients with platinum-resistant recurrent ovarian cancer	2	NCT01033292
**PARP**	AZD2281 (olaparib)	Study to assess the efficacy and safety of a PARP inhibitor for the treatment of BRCA-positive advanced ovarian cancer	2	NCT00494442
**PARP**	Cediranib Olaparib	Patients with ovarian cancer whose cancer worsened despite previously receiving a PARP inhibitor (such as olaparib)	–^*^	NCT02681237
**PARP**	Rucaparib Nivolumab	Treatment following response to frontline treatment in newly diagnosed ovarian cancer patient	3	NCT03522246
**ATR** **PARP**	AZD6738 Olaparib	ATARI trial test: ATR inhibitor drug AZD6738 and a PARP inhibitor drug olaparib in patients with relapsed gynaecological cancers with an abnormality in ARID1A gene	2	NCT04065269
**WEE1**	MK-1775 Carboplatin	Patients with p53 mutated epithelial ovarian cancer that have been treated with first line treatment (paclitaxel–carboplatin combination therapy) and that have shown early relapse (within 3 months)	2	NCT01164995
**ATR** **PARP**	AZD6738 Olaparib	Combination ATR and PARP inhibitor (CAPRI) trial with AZD 6738 and olaparib in recurrent ovarian cancer	2	NCT03462342
**ATR**	AZD6738 Paclitaxel	Refractory cancer patients who have failed to standard-of-care chemotherapy	1	NCT02630199
**ATR**	M6620 Avelumab Carboplatin	In participants with PARPi-resistant, recurrent, platinum-sensitive ovarian, primary peritoneal or fallopian tube cancer	2	NCT03704467
**ATR**	M6620 Gemcitabine	Patients with recurrent ovarian, primary peritoneal or fallopian tube cancer	2	NCT02595892
**ATR**	M6620 Topotecan	In small cell cancers and extrapulmonary small cell cancers	2	NCT02487095
**ATR**	M6620 Carboplatin, gemcitabine	Adult women with platinum-sensitive, recurrent high-grade serous or high-grade endometrioid ovarian, primary peritoneal or fallopian tube cancer	2	NCT02627443
**ATR**	M6620 Avelumab Nedisertib	DDR-deficient metastatic or unresectable solid tumors	1	NCT04266912
**ATR**	M6620 Carboplatin Gemcitabine	Patients with recurrent and metastatic ovarian, primary peritoneal or fallopian tube cancer	2	NCT02627443
**ATR**	BAY 1895344	Patients with advanced solid tumors and lymphomas	1	NCT03188965
**ATR** **PARP**	BAY 1895344 Niraparib	Advanced solid tumors and ovarian cancer	1	NCT04267939
**ATR**	VX-803 (M4344)	Women with recurrent ovarian cancer that has progressed while on a PARP inhibitor	1	NCT02278250
**ATR** **PARP**	VX-803 (M4344) Niraparib	Women with recurrent ovarian cancer that has progressed while on a PARP inhibitor	1	NCT04149145
**ATR** **PARP**	AZD 6738 Olaparib	HR-deficient patients with/without additional mutations in ATM, CHK-2, MRN (MRE11/NBS1/RAD50), CDKN2A/B and APOBEC	2	NCT02576444
**CHK1** **PARP**	Prexasertib (LY2606368) Olaparib	Solid tumors	1	NCT03057145
**CHK1**	Prexasertib (LY2606368)	BRCA1/2 mutation-associated breast or ovarian cancer, triple negative breast cancer and HGSOC	2	NCT02203513
**CHK1**	Prexasertib (LY2606368)	Patients with platinum-resistant or refractory ovarian cancer	2	NCT03414047

## DNA repair checkpoint inhibitors

### PARP

Inhibition of a DNA repair pathway sensitizes tumor cells with the BRCA 1/2 mutation to the DNA-damaging results of other chemotherapeutics. Given this, patients with BRCA^mut^ can respond more effectively to chemotherapy [8]. Therefore, the analysis of BRCA mutational status is crucial for therapeutic decisions [9]. An enhanced risk of ovarian cancer is related to DNA damage. Poly (ADP-ribose) polymerase (PARP) repairs ssDNA breaks. When these breaks are not repaired efficiently, which is the situation when PARP is blocked by the inhibitor, DSBs occur. DSBs are mainly repaired through two pathways: the HR pathway and the non-homologous end joining (NHEJ) pathway, although other mechanisms also exist. BRCA1 and BRCA2 participate in the DNA damage response, the network of interacting pathways that is essential for repair of genetic material. Both proteins are involved in the error-free repair of DSBs by HR in the S phase. BRCA1 signals DNA damage and ensures cell cycle regulation, while BRCA2 interacts and facilitates the loading and formation of RAD51 filaments on the damaged DNA strand. Tumors with impaired HR pathways lack an alternative DNA repair pathway [42, 43]. Mutation in BRCA1 or BRCA2 in HR-deficient cancer cells will lead to the repair of DSBs via error-prone repair pathways, accumulation of mutations and eventually cell death [44].

**Fig. 3:**
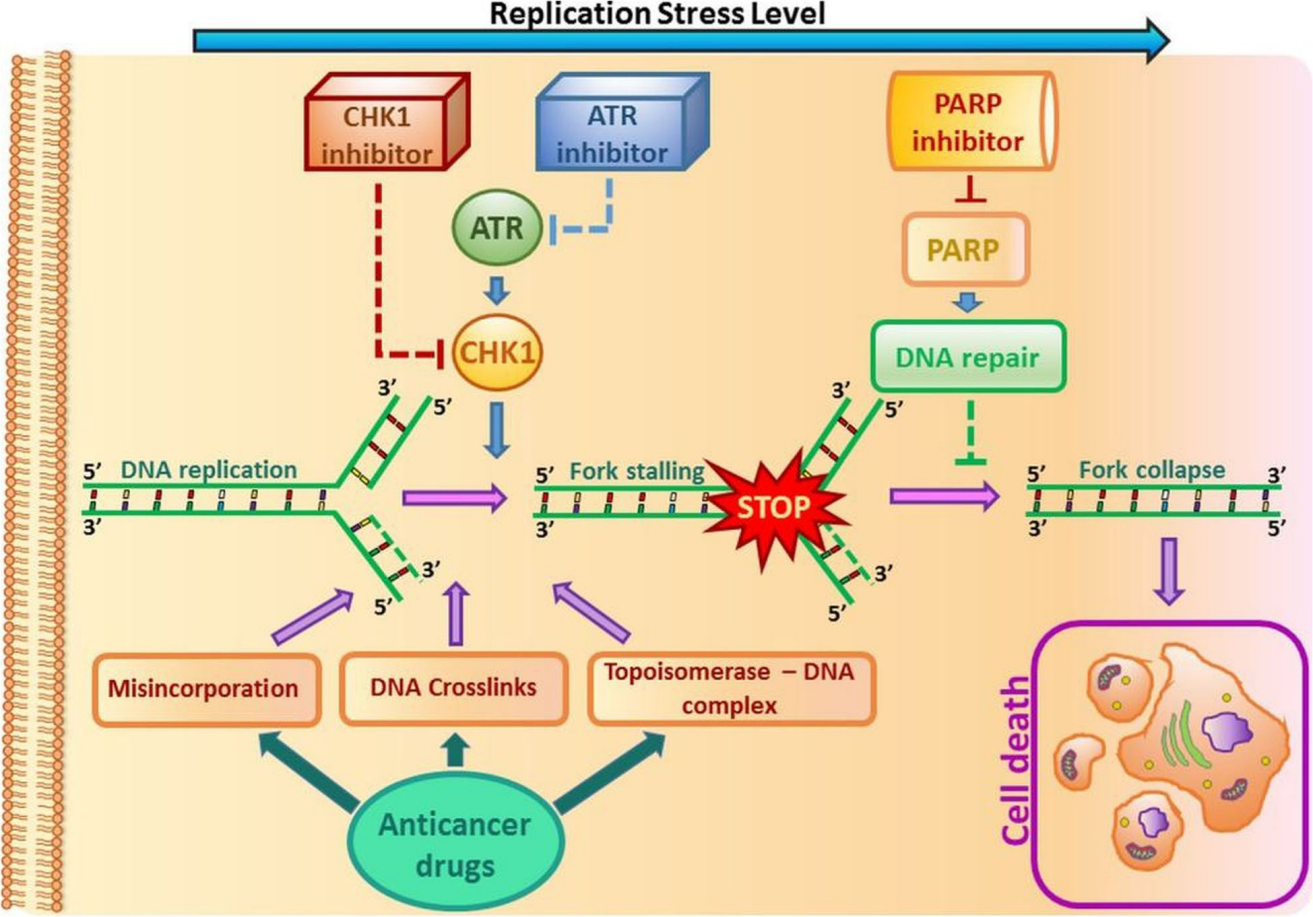
The role of PARP inhibitors in cancer therapy. PARP inhibitors are effective in HR-deficient cancer cells by the mechanism of synthetic lethality (left panel) and by PARP trapping (right panel). Other mechanisms of PARPi action are described in the text

Ovarian cancer cell lines with BRCA1 mutated and impaired HR (UWB1.289, SNU-251, OVCAR8) exhibit higher sensitivity towards PARPi, when compared with cells with wild-type or restored BRCA1 (SKOV3, A2780PAR and A2780CR) [45]. One group of compounds that have drawn intense research interest are PARPi inhibitors. In BRCA mutated cancer cells, PARP inhibition leads to tumor cell death, as a result of synthetic lethality. According to this assumption, PARPi blocks the base excision repair (BER) through F-Box DNA helicase 1 (Fbh1)-dependent Rad51 regulation [46]. Combination of olaparib with anticancer agents that disrupt HR repair represents an effective strategy to sensitize ovarian cancer cells. Synthetic lethality was defined classically in 1946 to describe a functional gene–gene relationship in *Drosophila*, in which two genes are nonlethal (viable) when inactivated alone but become lethal when inactivated together. Synthetic lethality is a consequence of the tendency of organisms to maintain buffering schemes that allow phenotypic stability despite genetic variation [47, 48]. Another mechanism of PARPi action involves PARPi binding to and trapping the PARP1 enzyme on chromatin (Fig. 3). Talazoparib, a PARPi, enhances trapping of PARP1 in DSBs, leading to decreased NHEJ and leukemia cell death [49, 50]. This agent is currently under clinical evaluation in patients with deleterious BRCA 1/2 mutation-associated ovarian cancer who have had prior PARP inhibitor treatment (NCT02326844). Moreover, DSBs can be resected by the microhomology-mediated end joining (MMEJ) pathway (also known as alternative non-homologous end-joining, Alt-NHEJ). MMEJ is independent from classical NHEJ and does not rely on NHEJ core factors such as Ku protein, DNA-dependent protein kinase (DNA-PK), or ligase IV. DNA polymerase θ (POLQ) plays an important role in this pathway. Efficient recruitment of POLQ depends on PARP1 [51, 52].

The first Food and Drug Administration (FDA)-approved PARP inhibitor was olaparib (Lynparza). In 2005, the first two publications demonstrating the substantial sensitivity of BRCA-deficient cell lines to inhibition of PARP led to an unprecedented and swift implementation of PARP inhibitors in clinical practice [53]. Several PARP inhibitors, including olaparib, niraparib, veliparib, rucaparib and talazoparib, are being tested in clinical trials, and olaparib, niraparib and rucaparib (NCT03522246) have been registered for use in a clinical setting. In 2014, olaparib gained European Medicines Agency (EMA) approval to treat advanced EOC in monotherapy of patients with germline BRCA^mut^ who had received and did not respond to at least three lines of chemotherapy [54]. A phase II clinical trial revealed that olaparib significantly increased the effectiveness of standard treatment with a combination of carboplatin and paclitaxel. Progression-free survival was longer by around 3 months in the olaparib with chemotherapy group compared with chemotherapy alone. In the USA in 2018, the FDA-approved olaparib for the maintenance treatment of patients with BRCA^mut^ advanced EOC who are in complete or partial response to first-line platinum-based chemotherapy [55]. Two more PARP inhibitors, rucaparib (Rubraca) and niraparib (Zejula), approved by the FDA, are a promising class of agents for targeted EOC therapy [56]. Research is ongoing into the use of iniparib in combination with carboplatin and gemcitabine in the treatment of patients with platinum-resistant recurrent ovarian cancer (NCT01033292). PARP inhibitors are usually combined with other drugs to increase the therapeutic effect. A combination of two investigational drugs, cediranib and olaparib, was evaluated in patients with ovarian cancer whose cancer worsened despite previously receiving a PARP inhibitor such as olaparib (NCT02681237).

Clinical trials have shown PARPi response rates of around 40% in women who carry a mutation in the BRCA1/2 genes (NCT00494442). However, PARPi is responsible for tumor suppression, but not for complete tumor regression [57]. Although olaparib is a step forward in the treatment of BRCA1/2-deficient tumors, resistance to PARP inhibitors is unfortunately a common phenomenon. Rare complete responses are seen with PARPi monotherapy in clinical practice [58]. BRCA1/2-deficient tumor cells can become resistant to PARP inhibitors by restoring HR repair and/or by stabilizing replication forks. PARPi-induced drug resistance mechanisms have focused also on heat shock protein 90 (HSP90)-mediated stabilization of BRCA1^mut^, RAD51 upregulation, loss of REV7 or promotion of alternative error-prone NHEJ DNA repair [59, 60]. Furthermore, a BRCA2-mutated ovarian cancer cell line, with sensitivity to both platinum and PARPi, regained the BRCA2 function by secondary mutation after treatment with cisplatin and PARPi [61]. In addition, an acquired low level of expression of PARP1 may be a cause of resistance to PARPi in patient-derived tumor xenograft models [62]. Moreover, as a result of PARP1 inhibition, cancer cells may upregulate the HR repair pathway and increase RAD51 expression to maintain cell viability [63]. The formation of RAD51 foci was observed together with PARPi resistance in patient-derived xenograft models, as well as patient-derived samples carrying the BRCA^mut^ [64]. On the other hand, increased expression of the ATP-binding cassette sub-family B member 1 (ABCB1) (also known as multidrug resistance protein 1 [MDR1]), which encodes the membrane drug transporter P-glycoprotein, is a well-described mechanism of resistance to doxorubicin, paclitaxel and related taxane drugs. ABCB1-mediated resistance to PARPi is a novel finding in ovarian cancer [65]. Nonetheless, loss of BRCA1/2 factors determines PARPi sensitivity. MiR-493-5p may induce platinum and PARPi resistance, specifically in the cells with BRCA2 mutation [66]. Hence, there is an urgent need to develop new, more effective strategies.

All drugs, including olaparib, have potential side effects. Most side effects of olaparib were of low grade, with anaemia and neutropenia being the most common, in the SOLO1 trail [67]. On the other hand, cardiac adverse effects are the leading cause of discontinuation of clinical trials and withdrawal of drugs from the market. There are reports suggesting that olaparib is a cardioprotective agent against doxorubicin-induced cardiomyopathy [68] and that it protects cardiomyocytes against oxidative stress [69].

### ATR

**Fig. 4:**
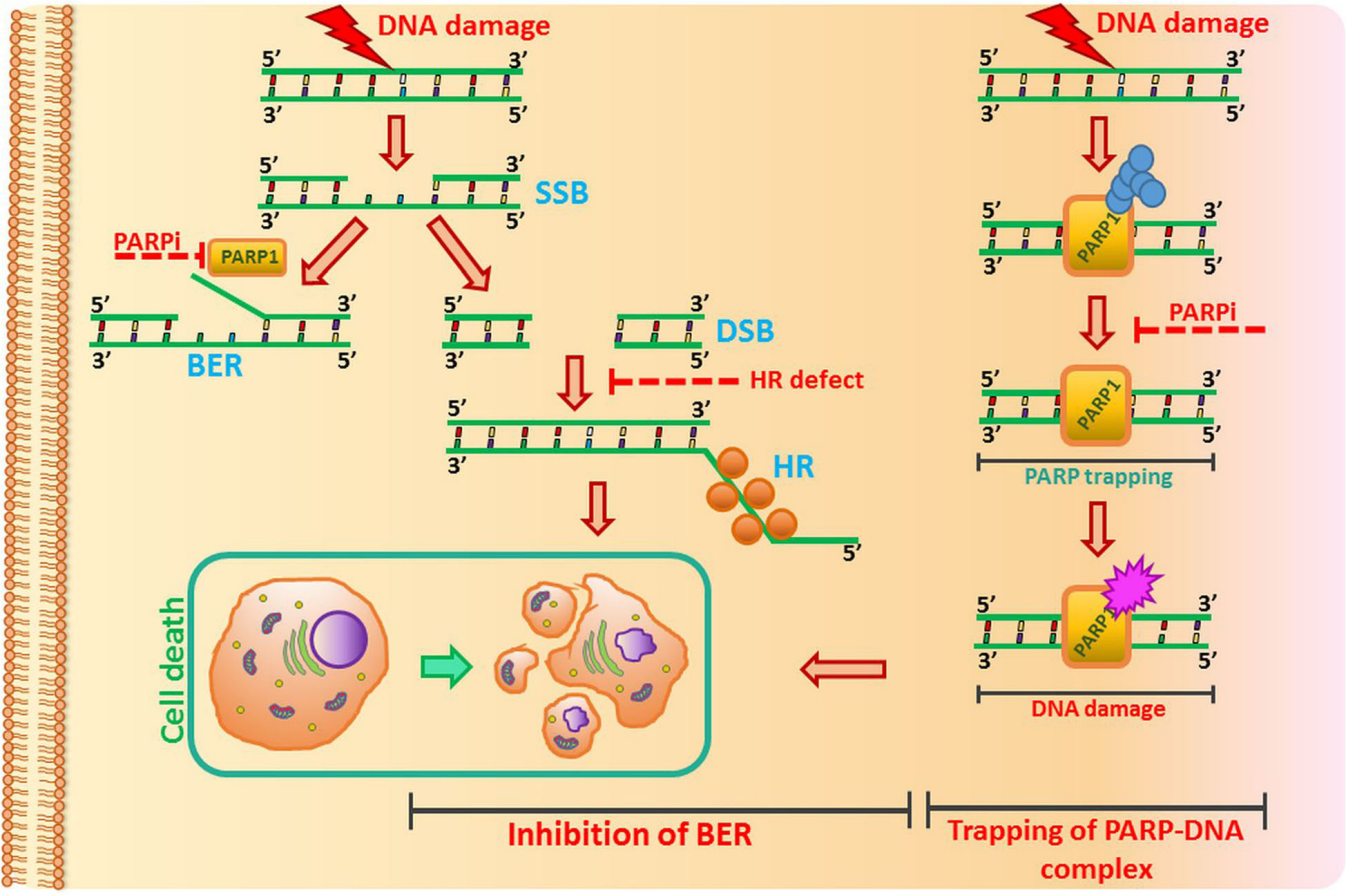
Participation of ATR in maintenance of genome stability. DNA double strand breaks or replication stress induce an ATR protein response. ATR is recruited to regions of ssDNA replication protein A (RPA) through its partner, ATR-interacting protein (ATRIP). Subsequently, RAD9–RAD1-hus1 (9-1-1 complex) and DNA topoisomerase 2 binding protein 1 (TOPBP1) are incorporated, leading to ATR activation. ATR–ATRIP recruitment results in CHK1 activation. This process is mediated by Claspin, Timeless and Tipin, which form a complex at replication forks. In the event of large areas of DNA damage or no repair, the replication fork stops, origin suppression occurs and the cell cycle is stopped. ATR/CHK1 blockade prevents DNA damage-induced cell-cycle arrest, resulting in inappropriate entry into mitosis, chromosome aberrations, unequal partitioning of the genome, and apoptosis

ATM and ATR kinases are two master regulators of DNA damage responses. ATR, a serine/threonine-protein kinase belonging to the phosphatidylinositol 3-kinase-related kinase (PIKK) family of proteins, is a key regulator of the DNA replication stress response (RSR) and DNA-damage activated checkpoints [70]. As ATR is a master regulator of the DDR, this finding underscores the relevance of DDR as a new therapeutic target in ovarian cancer therapy. ATR is activated in response to a broad spectrum of DNA damage, such as single- and double-stranded DNA, and also adducts, cross-links and inhibits DNA polymerase, while ATM is primarily activated in response to DNA double-strand breaks [71–73]. Moreover, ATM is responsible for the phosphorylation of checkpoint kinase 2 (CHK2) and ATR phosphorylates checkpoint kinase 1 (CHK1) [74]. CHK1 activation is mediated by Claspin (CLSPN), Timeless and Tipin [75]. The ATR lies upstream of CHK1 and phosphorylates numerous factors including Werner syndrome ATP-dependent helicase (WRN), SWI/SNF-related matrix-associated actin-dependent regulator of chromatin sub-family A-like protein 1 (SMARCAL1), and Fanconi anaemia complementation group I (FANCI), which may help preserve replication fork stability and control cell-cycle progression [76–78]. The direct substrates of ATR include RPA, MCM2, p53 and many other factors that play roles in replication fork progression, DNA repair and control of the cell cycle [79, 80]. Additionally, ATR substrates control protein modification, transcriptional regulation and developmental processes [81]. ATR has a crucial role in stabilizing genomic integrity throughout the cell cycle and is therefore essential for cell survival [82] (Fig. 4). ATR controls cell cycle arrest from S to G2 phases [83]. Furthermore, ATR plays a role in the G2/M phase checkpoint [84]. In cells with TP53 mutation, it leads to checkpoint-defective cells, and the inhibition of ATR is lethal [85, 86]. Dysregulation of ATR disrupts a broad range of cellular processes [16]. Recent reports suggest that cells with impaired HR activities due to mutations in TP53 gene or specific DNA repair proteins are specifically sensitive to ATR inhibitors [86–89]. However, the underlying mechanisms of ATR inhibition monotherapy on ATM status remain unclear [84].

ATR inhibitors have the potential to show preferential cell killing of tumor cells where ATM is defective or where replicative stress is high. In recent years, ATR inhibitors with far greater potency and selectivity have been developed as drug-like agents. Many ATR inhibitors, such as NU6027, ETP-46464 and VX-970, also inhibit CDK2, mTOR, ATM and PIK3 kinases [90, 91]. VX-970/M6620 is an analogue of VE-821 from the aminopyrazine series with a marked increase in ATR enzyme cytotoxicity (IC_50_ = 0.019 μM) in HCT116 cells. VX-970 is selective versus DNA-PK, mTOR, PI3Kγ and 50 unrelated protein kinases [92]. One of the most promising is AZD6738, which is a potent and selective sulfoximine morpholinopyrimidine ATR inhibitor.

AZD6738 is currently used as a monotherapy and in combination with gemcitabine (NCT02595892), gemicitabine and carboplatin (NCT02627443), avelumab and nedisertib (NCT04266912), paclitaxel (NCT02630199) and radiotherapy (NCT02223923). VE-821, another identified ATR inhibitor, sensitized OVCAR-8, SKOV-3 and PEO1 ovarian cancer cell lines to cisplatin, topotecan and veliparib [93, 94]. Additionally, cisplatin or topotecan, combined with MK-877 (CHK1i) or VE-821, reduced the G2/M phase accumulations [94]. Currently, clinical trials are ongoing on M6620 in combination with gemcitabine, avelumab (NCT03704467), topotecan (NCT02487095) and carboplatin (NCT02627443) in ovarian cancer. On the other hand, VX-970 is the most effective in combination with platinum agents or melphalan [95]. BAY 1895344 is a new potent ATR inhibitor developed by Bayer. BAY 1895344 is being studied in cancers with DNA repair deficiency. The in vivo antitumor efficacy of BAY 1895344 in combination with carboplatin was investigated in the IGROV-1 ovarian cancer model [96]. BAY 1895344 is currently under clinical investigation in patients with advanced solid tumors and lymphomas (NCT03188965). VX-803 (M4344), on the other hand, is now in clinical trials as a monotherapy or in combination with carboplatin for advanced solid tumors (NCT02278250), and in combination with niraparib against ovarian cancer (NCT04149145).

### CHK1

CHK1 is a serine/threonine kinase, which responds to DNA damage and replication stress and therefore regulates mitotic progression. ATR and CHK1 share the same pathway; it is possible that the antitumor properties of ATR inhibitors may not differ significantly from those of CHK1 inhibitors currently tested in clinical trials. Moreover, ATR controls many other proteins, not only CHK1, as described in the previous section, suggesting that it is responsible for controlling additional cell responses. Nonetheless, CHK1 can be autophosphorylated and thus activated, independently of ATR [97].

Activation of CHK1 and its downstream effectors leads to an array of coordinated activities that include reduced new origin firing, delay of cell cycle progression and restoration of the stalled replication forks. The phosphorylation of CHK1 at Ser317 and Ser345 after checkpoint signaling is regulated by ATR, and thus, phosphorylation at Ser345 fosters relocalization or retention of the protein in the nucleus [98]. Translocation of the protein from the cytoplasm to the nucleus causes the activation of the G2/M checkpoint and regulates cell cycle progression [99] by inactivating CDC25 phosphatases (CDC25A and CDC25C), which would otherwise activate the CDKs, responsible for the G2/M transition [100]. Cancer cells with p53 mutation cannot activate the G1/S checkpoint and relies only on the S and G2/M checkpoints, both controlled by CHK1 [101]. This suggests that patients with p53-mutated tumors could benefit from the treatment based on CHK1 inhibition [102]. CHK1 also phosphorylates BRCA2 and RAD51 proteins [103]. Therefore, CHK1 inhibition renders the cells to be more sensitive to DNA damage [93]. As previously mentioned, CHK1 may also be autophosphorylated at Ser296 [104]; thus, once activated, it may restrain replication catastrophe in S-phase, even in the presence of an ATRi [97].

Several CHK1 inhibitors are known, including compounds in research phase I (AZD7762, PF-477736, GDC-0425 and GDC-0575) or in phase II (LY2603618 and LY2606368) of trials in combination with gemcitabine, irinotecan, pemetrexed and cytarabine [105, 106]. V158411 inhibited CHK1 and CHK2 and abolished DNA damage-induced S- and G2-phase checkpoints. The in vitro cytotoxicity of gemcitabine, cisplatin, SN38 and camptothecin was potentiated by V158411 in TP53-deficient, but not in TP53-proficient, human tumor cell lines [107]. Research has also been conducted on Prexasertib (LY2606368), which is a small ATP-competitive selective inhibitor of CHK1 and CHK2. Prexasertib blocks the autophosphorylation of and subsequently activates the CHK proteins, which regulate the activity of phase-M inducer cyclin-dependent kinases and phosphatases [108]. Prexasertib is currently in phase 1 and 2 clinical trials and was tested either as a single agent (NCT02203513, NCT03414047) or in combination with olaparib in 14 clinically annotated and molecularly characterized luciferized HGSOC patient-derived xenograft (PDX) models, and in a panel of ovarian cancer cell lines. The ability of prexasertib to impair HR repair and replication fork stability was assessed. Thirteen models were resistant to olaparib monotherapy, including four carrying a BRCA1 mutation [102]. Another example is MK-8776 (formerly, SCH900776, a pyrazolopyrimidine derivative), a highly selective CHK1 inhibitor [79]. MK-8776 is approximately 500 times more selective for CHK1 than for CHK2. MK-8776 and LY 2603618 sensitized cells only to gemcitabine [93, 94]. UCN-01, another CHK1 inhibitor, also exhibits a radiosensitivity effect; however, MK-8776 demonstrated more pronounced effects with lower cytotoxicity [80]. Even in the absence of DNA damage caused by external agents, CHK1/2 inhibition yielded DNA damage and mitotic catastrophe preclinically in tumors with DNA repair dysfunction [109]. Moreover, in cells lacking BRCA1, CHK1 is necessary for the repair of endogenous DNA damage; therefore, inhibition of this protein had an anti-proliferative effect on the cells [102]. Several potential mechanisms for sensitization by CHK1 inhibition have been proposed, including inhibition of repair systems for DSBs, spindle assembly checkpoint (SAC) activation, promotion of premature mitosis, and mitotic catastrophe (MC). However, it remains unclear how CHK1 inhibition triggers sensitization in ovarian cancer cells.

## Synergy in cell killing based on ATR/CHK1 and PARP inhibitors

BRCA1/2 proteins play an important role in the protection of stalled replication forks. This is controlled by the ATR/CHK1 checkpoint kinase pathway. Impaired ATM kinase function activates ATR. This system of mutual taking of functions in the cell has been used to fight cancer. By binding to reversed forks, the BRCA1/2 proteins play a critical role in protecting the cell from genomic instability. On the other hand, ATR/CHK1 blockade prevents DNA damage-induced cell-cycle arrest, resulting in inappropriate entry into mitosis, chromosome aberrations, unequal partitioning of the genome, and apoptosis [93]. The ATR/CHK1 pathway stabilizes replication forks and prevents their collapse into DNA double-strand breaks. Thus, inhibition of ATR/CHK1 is expected to increase reliance on HR to reorganize the replication fork structure and complete replication. ATR inhibition is lethal with numerous cancer-associated changes, including oncogenic stress (oncogenic RAS mutations, MYC and G1/S-specific cyclin-E1 (CCNE1) overexpression), deficiencies in DNA repair (TTP53, BRCA1/2, partner and localizer of BRCA2 (PALB2) and ATM loss) and other defects [110]. Several studies have shown that some tumors are even more sensitive to combinations of ATR inhibitors with inhibitors of other repair proteins such as PARP and CHK1. Inhibiting these proteins alone may be insufficient to cause cell death, so it may be necessary to apply PARPi and cell cycle checkpoint inhibitors as combination therapies [57].

Clinical trials are currently underway using the combination of AZD6738 and olaparib in recurrent ovarian cancer (NCT03462342) and in gynaecological cancers with ARId1A loss or no loss (NCT04065269). An extensive signal-searching study is being conducted. HR-deficient patients with/without additional mutations in ATM, CHK-2, MRN (MRE11/NBS1/RAD50), CDKN2A/B and APOBEC will be treated with olaparib or olaparib and AZD6738 (NCT02576444). Moreover, patients with advanced solid tumors and ovarian cancer will be treated with BAY1895344 in combination with niraparib **(**NCT04267939). The investigators are combining LY2606368 (CHK1i) with olaparib (NCT03057145) in patients with solid tumors. Moreover, NU6027 (CHK1i) in combination with a PARP inhibitor was shown to attenuate G2/M arrest and was synthetically lethal in the MCF7 cell line [87].

## New platforms for optimizing ovarian cancer therapy under replication stress

New models for preclinical ovarian cancer have been sought for a long time. The resolve to achieve this was strengthened with the decision by the National Cancer Institute (NCI) to retire the NCI-60, a panel of 60 human cancer cell lines grown in culture, from its drug screening programme [111]. Cell lines and cell-line-derived xenografts (CDXs) are still the most commonly used basic research models in ovarian cancer. Recently, scientific interest has increasingly focused on the cancer microenvironment. Given this, preclinical modelling of individual cancers should be included in the framework of personalized medicine. Currently, attention is focused on promising new research models including patient-derived xenografts (PDX) and organoids.

PDX maintain the characteristics of the patient’s original tumor, including histology, mutational status and gene expression, through multiple passages in mice. PDX also exhibit a similar response to standard chemotherapy to that demonstrated in the patient. They are a copy of the xenograft model in which fresh tumor tissue is obtained directly from patients and implanted into an immunodeficient mice lacking a human immune system [57]. Different types of mice (e.g. NCG, NOD-Prkdc^em26Cd52^ IL2rg^em26Cd22^/Gp t; NSG, NOD-SCID IL2Rγ^−/−^) have been used in PDX models of ovarian cancer. Olaparib, MK8776 or AZD6738 were used to treat ovarian tumors with BRCA2^mut^ obtained with PDX [112]. Moreover, PDX has been applied to study the olaparib response predicted on the basis of HR gene analysis. The homogenate was injected subcutaneously into the lower dorsal flank or axilla of the NCG mice and DDR mutation analysis of PDX cases was carried out [113]. A PDX model (NSG mice) was also derived from BRCA-mutant HGSOCs exhibiting solid, pseudoendometrioid and transitional cell carcinomas. It exhibited higher levels of phosphorylated CHK1 than BRCA-intact HGSOC. Using PET imaging, studies have shown that PARP inhibitor treatment results in tumor suppression but not complete tumor regression, similar to the response observed in clinical settings [57]. The response of 12 HGSOCs (PDX; NSG mice) to the PARPi rucaparib was measured, with dose-dependent responses observed in chemo-naive BRCA1/2-mutated PDX and no responses in PDX lacking DNA repair pathway defects. Among BRCA1-methylated PDX, silencing of all BRCA1 copies predicted the rucaparib response, whereas heterozygous methylation was correlated with resistance (ARIEL2 part 1 trial) [114].

Until now, PDX models could only be established in immunocompromised mouse strains. Currently, to assess immunotherapy, research is being carried out on PDX to which autologous transfer of patient-specific tumor infiltrating lymphocytes (TILs) has been performed [115].

Another approach in personalized medicine involves organoid cultures of patient-derived tumors. These are three-dimensional (3D) constructs that represent an excellent preclinical model for human tumors that has cancer homology and has been gradually applied to gene analysis, drug screening and other types of research; they facilitate the translation from basic cancer research to clinical practice [116]. Researchers have also begun to apply organoids in studies of ovarian cancer. Several teams have confirmed that patient-derived organoids closely resemble the original gynecologic tumors, and thereby may serve as a promising resource for preclinical studies [117, 118]. Hans Clevers’ team collected 56 organoids from 32 patients, representing all the main subtypes of OC, and confirmed that homologous recombinant (HR) defective cells are sensitive to PARP inhibitors, which are also present in the ovarian cancer organoid [117]. Another team developed a HGSOC organoid and used it for functional analysis of DNA repair and prediction of patients’ clinical response to DNA repair inhibitors [118]. By studying the HR and cross-protection defects of 33 HGS-like organoids in 22 patients, it was confirmed that the functional defects of HR in the organoid are related to the sensitivity of PARP inhibitors regardless of the mutation status of DNA repair genes. In addition, the functional defects in cross-protection of replication are related to the sensitivity to carboplatin, CHK1, and ATR inhibitors. These findings indicate that genome analysis and organ-like function testing can identify targeted DNA damage and repair defects. The OC organoid can be used for DNA repair analysis and therapeutic sensitivity testing, which can immediately evaluate target defects in maternal tumors and provide appropriate treatment options [118].

Western blot analysis of 33 organoid cultures showed that prexasertib increases DNA damage, indicated by increased expression of γH2AX, and increased replication stress, as indicated by increased phosphorylated RPA (pRPA). Prexasertib activates the ATR pathway in both fork-unstable and fork-stable lines, as shown by the increased phosphorylation of the ATR targets KAP1 (pKAP1) and CHK1 (pCHK1) [119]. The elevated pCHK1 level is a pharmacodynamic marker of CHK1 inhibition by prexasertib. Prexasertib stimulates a tumor in terms of sensitivity to other DNA repair agents, blocking the ATR/CHK1 pathway, thereby increasing replication stress [120]. In addition, other factors that increase replication stress may interact with prexasertib and promote fork instability and cancer cell death. For example, regardless of the genetic status, a stalled fork protection defect was present in 61% of the organoid lines tested, and this defect was associated with carboplatin, prexasertib, and VE-822 sensitivity. In contrast, only 6% of organoid lines tested had a functional HR defect and PARPi sensitivity. Overall, this suggests that stalled fork protection defects are more common than HR defects and have a larger array of specific therapies [118].

## Conclusions

The first generation of ATR and CHK1 inhibitors has been shown to sensitize ovarian tumors to DNA-damaging agents that primarily induce replicative stress as their mechanism of action. The analysis of BRCA mutational status is still the first step in designing individualized strategies for the management of patients with ovarian cancer. Inhibition of ATR or CHK1 as a monotherapy or in combination with DNA-damaging chemotherapy drugs or PARP inhibitors is being tested in early-phase clinical trials in gynaecological cancers. ATR inhibitors, such as M6620 and AZD6738, give a very good prognosis. Phase 2 combination trials are ongoing. In turn, two of the best studied CHK1 inhibitors are MK-8776 and prexasertib. Preclinical observations indicate that the synthetic lethality of ATR or CHK1 inhibitors in ATM-deficient cancers may be a new opportunity for effective ovarian cancer therapy.

We also need to understand the long-term tolerability of the various PARP inhibitors and the mechanisms leading to the development of multidrug resistance. Moreover, mutations and damage in ATM or p53 genes are therapeutic opportunities for inhibitors involved in replication stress. Despite learning about a dozen or so genes that lead to synthetic lethality with PARP, new ones are still being sought. The development of new in vivo model systems such as PDX and organoids will facilitate the optimization of ovarian cancer therapy. Clustered regularly interspaced short palindromic repeats (CRISPR)-directed Cas9-mediated endonuclease activity disrupts specific genetic sequences in the genome and is a new tool for finding therapeutic goals. In this way, the sequence of C12ofr5 was identified, which is a gene that encodes a metabolic regulator, TP53-induced glycolysis and apoptosis regulator (TIGAR). Downregulation of TIGAR results in enhanced cytotoxic effects of olaparib [121]. Only further deepening of the knowledge about genes involved in DNA repair and blocking all restoration options in the pathway can lead to definitive ovarian cancer cell death.

## Data Availability

All data and materials supporting the conclusions of this study have been included within the article.

## Abbreviations

ABCB1: ATP-binding cassette sub-family B member 1
ATM: Ataxia telangiectasia mutated protein
ATR: Ataxia telangiectasia and Rad3-related protein
ATRIP: ATR-interacting protein
BER: Base excision repair
BRCA: Breast cancer gene
CDK: Cyclin-dependent kinases
CHK1: Checkpoint kinase 1
DDR: DNA damage response
DNA-PK: DNA-dependent protein kinase
EOC: Epithelial ovarian cancer
HGSOCs: High-grade serous ovarian carcinomas
HR: Homologous recombination
MMEJ: Microhomology-mediated end joining
NHEJ: Non-homologous end joining
PARP: Poly (ADP-ribose) polymerase
PCNA: Proliferating cell nuclear antigen
PIKKs: Phosphatidylinositol 3-kinase-related kinases
POLQ: DNA polymerase θ
RPA: Replication protein A
RSR: Replication stress response
TopBP1: DNA topoisomerase 2-binding protein 1
WEE1: Tyrosine kinase

## References

[1] Momenimovahed Z, Tiznobaik A, Taheri S, Salehiniya H (2019). Ovarian cancer in the world: epidemiology and risk factors. Int J Womens Health..

[2] Budiana ING, Angelina M, Pemayun TGA (2019). Ovarian cancer: pathogenesis and current recommendations for prophylactic surgery. J Turk Ger Gynecol Assoc..

[3] McGee J, Peart TM, Foley N, Bertrand M, Prefontaine M, Sugimoto A (2019). Direct genetics referral pathway for high-grade serous ovarian cancer patients: the “opt-out” process. J Oncol..

[4] Siegel RL, Miller KD, Jemal A (2019). Cancer statistics, 2019. CA Cancer J Clin..

[5] Koshiyama M, Matsumura N, Konishi I (2014). Recent concepts of ovarian carcinogenesis: type I and type II. Biomed Res Int..

[6] Cancer Genome Atlas Research N (2011). Integrated genomic analyses of ovarian carcinoma. Nature..

[7] Konstantinopoulos PA, Ceccaldi R, Shapiro GI, D'Andrea AD (2015). Homologous recombination deficiency: exploiting the fundamental vulnerability of ovarian cancer. Cancer Discov..

[8] Huang YW (2018). Association of BRCA1/2 mutations with ovarian cancer prognosis: an updated meta-analysis. Medicine (Baltimore)..

[9] Gadducci A, Guarneri V, Peccatori FA, Ronzino G, Scandurra G, Zamagni C (2019). Current strategies for the targeted treatment of high-grade serous epithelial ovarian cancer and relevance of BRCA mutational status. J Ovarian Res..

[10] Dasari S, Tchounwou PB (2014). Cisplatin in cancer therapy: molecular mechanisms of action. Eur J Pharmacol..

[11] Komiyama S, Nagashima M, Taniguchi T, Rikitake T, Morita M. Bevacizumab plus direct oral anticoagulant therapy in ovarian cancer patients with distal deep vein thrombosis. Clin Drug Investig. 2019. 10.1007/s40261-019-00757-w 30737671

[12] Park GB, Ko HS, Kim D (2017). Sorafenib controls the epithelialmesenchymal transition of ovarian cancer cells via EGF and the CD44HA signaling pathway in a cell typedependent manner. Mol Med Rep..

[13] DeVorkin L, Hattersley M, Kim P, Ries J, Spowart J, Anglesio MS (2017). Autophagy inhibition enhances sunitinib efficacy in clear cell ovarian carcinoma. Mol Cancer Res..

[14] Lokadasan R, James FV, Narayanan G, Prabhakaran PK (2016). Targeted agents in epithelial ovarian cancer: review on emerging therapies and future developments. Ecancermedicalscience..

[15] Liu R, Guo H, Lu S (2018). MiR-335-5p restores cisplatin sensitivity in ovarian cancer cells through targeting BCL2L2. Cancer Med..

[16] Wang J, He J, Su F, Ding N, Hu W, Yao B (2013). Repression of ATR pathway by miR-185 enhances radiation-induced apoptosis and proliferation inhibition. Cell Death Dis..

[17] Rybaczek D (2016). Hydroxyurea-induced replication stress causes poly (ADP-ribose) polymerase-2 accumulation and changes its intranuclear location in root meristems of *Vicia faba*. J Plant Physiol..

[18] Murai J (2017). Targeting DNA repair and replication stress in the treatment of ovarian cancer. Int J Clin Oncol..

[19] Beggs R, Yang ES (2019). Targeting DNA repair in precision medicine. Adv Protein Chem Struct Biol..

[20] Harrington KJ. Chemotherapy and targeted agents. Maxillofacial Surgery. 2017:339–54.

[21] Potapova TA, Daum JR, Byrd KS, Gorbsky GJ (2009). Fine tuning the cell cycle: activation of the Cdk1 inhibitory phosphorylation pathway during mitotic exit. Mol Biol Cell..

[22] Perry JA, Kornbluth S (2007). Cdc25 and Wee1: analogous opposites?. Cell Div..

[23] Brandsma I, Fleuren EDG, Williamson CT, Lord CJ (2017). Directing the use of DDR kinase inhibitors in cancer treatment. Expert Opin Investig Drugs..

[24] Schmid BC, Oehler MK (2014). New perspectives in ovarian cancer treatment. Maturitas..

[25] Westin SN, Sood AK, Coleman RL. Targeted therapy and molecular genetics. Clin Gynecol Oncol. 2018:470–92.

[26] Moiseeva TN, Qian C, Sugitani N, Osmanbeyoglu HU, Bakkenist CJ (2019). WEE1 kinase inhibitor AZD1775 induces CDK1 kinase-dependent origin firing in unperturbed G1- and S-phase cells. Proc Natl Acad Sci U S A..

[27] Azenha D, Lopes MC, Martins TC (2019). Claspin: from replication stress and DNA damage responses to cancer therapy. Adv Protein Chem Struct Biol..

[28] Leijen S, van Geel RM, Sonke GS, de Jong D, Rosenberg EH, Marchetti S (2016). Phase II study of WEE1 inhibitor AZD1775 plus carboplatin in patients with TP53-mutated ovarian cancer refractory or resistant to first-line therapy within 3 months. J Clin Oncol..

[29] Ivy SP, Kunos CA, Arnaldez FI, Kohn EC (2019). Defining and targeting wild-type BRCA high-grade serous ovarian cancer: DNA repair and cell cycle checkpoints. Expert Opin Investig Drugs..

[30] Marechal A, Zou L. DNA damage sensing by the ATM and ATR kinases. Cold Spring Harb Perspect Biol. 2013;5(9). 10.1101/cshperspect.a012716 PMC3753707 24003211

[31] Roos WP, Kaina B (2013). DNA damage-induced cell death: from specific DNA lesions to the DNA damage response and apoptosis. Cancer Lett..

[32] Sun XL, Jiang H, Han DX, Fu Y, Liu JB, Gao Y (2018). The activated DNA double-strand break repair pathway in cumulus cells from ageing patients may be used as a convincing predictor of poor outcomes after in vitro fertilization-embryo transfer treatment. PLoS One..

[33] Beyaert M, Starczewska E, Perez ACG, Vanlangendonck N, Saussoy P, Tilman G (2017). Reevaluation of ATR signaling in primary resting chronic lymphocytic leukemia cells: evidence for pro-survival or pro-apoptotic function. Oncotarget..

[34] Rybaczek D, Kowalewicz-Kulbat M (2011). Premature chromosome condensation induced by caffeine, 2-aminopurine, staurosporine and sodium metavanadate in S-phase arrested HeLa cells is associated with a decrease in Chk1 phosphorylation, formation of phospho-H2AX and minor cytoskeletal rearrangements. Histochem Cell Biol..

[35] Bian X, Lin W. Targeting DNA replication stress and DNA double-strand break repair for optimizing SCLC rreatment. Cancers (Basel). 2019;11(9). 10.3390/cancers11091289 PMC6770306 31480716

[36] Nie Y, Lang T (2017). The interaction between ATRIP and MCM complex is essential for ATRIP chromatin loading and its phosphorylation in mantle cell lymphoma cells. Pharmazie..

[37] Fukumoto Y, Takahashi K, Suzuki N, Ogra Y, Nakayama Y, Yamaguchi N (2018). Casein kinase 2 promotes interaction between Rad17 and the 9-1-1 complex through constitutive phosphorylation of the C-terminal tail of human Rad17. Biochem Biophys Res Commun..

[38] Chen Y, Li J, Cao F, Lam J, Cheng CC, Yu CH (2018). Nucleolar residence of the seckel syndrome protein TRAIP is coupled to ribosomal DNA transcription. Nucleic Acids Res..

[39] Post SM, Tomkinson AE, Lee EY (2003). The human checkpoint Rad protein Rad17 is chromatin-associated throughout the cell cycle, localizes to DNA replication sites, and interacts with DNA polymerase epsilon. Nucleic Acids Res..

[40] Abraham RT (2001). Cell cycle checkpoint signaling through the ATM and ATR kinases. Genes Dev..

[41] Abbotts R, Thompson N, Madhusudan S (2014). DNA repair in cancer: emerging targets for personalized therapy. Cancer Manag Res..

[42] Lord CJ, Ashworth A (2013). Mechanisms of resistance to therapies targeting BRCA-mutant cancers. Nat Med..

[43] Pitroda SP, Pashtan IM, Logan HL, Budke B, Darga TE, Weichselbaum RR (2014). DNA repair pathway gene expression score correlates with repair proficiency and tumor sensitivity to chemotherapy. Sci Transl Med.

[44] Rajawat J, Shukla N, Mishra DP (2017). Therapeutic targeting of poly(ADP-Ribose) polymerase-1 (PARP1) in cancer: current developments, therapeutic strategies, and future opportunities. Med Res Rev..

[45] Baloch T, Lopez-Ozuna VM, Wang Q, Matanis E, Kessous R, Kogan L (2019). Sequential therapeutic targeting of ovarian cancer harboring dysfunctional BRCA1. BMC Cancer..

[46] Ronson GE, Piberger AL, Higgs MR, Olsen AL, Stewart GS, McHugh PJ (2018). PARP1 and PARP2 stabilise replication forks at base excision repair intermediates through Fbh1-dependent Rad51 regulation. Nat Commun..

[47] Bryant HE, Schultz N, Thomas HD, Parker KM, Flower D, Lopez E (2005). Specific killing of BRCA2-deficient tumours with inhibitors of poly(ADP-ribose) polymerase. Nature..

[48] Farmer H, McCabe N, Lord CJ, Tutt AN, Johnson DA, Richardson TB (2005). Targeting the DNA repair defect in BRCA mutant cells as a therapeutic strategy. Nature..

[49] Valdez BC, Li Y, Murray D, Liu Y, Nieto Y, Champlin RE (2018). Combination of a hypomethylating agent and inhibitors of PARP and HDAC traps PARP1 and DNMT1 to chromatin, acetylates DNA repair proteins, down-regulates NuRD and induces apoptosis in human leukemia and lymphoma cells. Oncotarget..

[50] Robert C, Nagaria PK, Pawar N, Adewuyi A, Gojo I, Meyers DJ (2016). Histone deacetylase inhibitors decrease NHEJ both by acetylation of repair factors and trapping of PARP1 at DNA double-strand breaks in chromatin. Leuk Res..

[51] Gourley C, Balmana J, Ledermann JA, Serra V, Dent R, Loibl S (2019). Moving from poly (ADP-Ribose) polymerase inhibition to targeting DNA repair and DNA damage response in cancer therapy. J Clin Oncol..

[52] Dai CH, Chen P, Li J, Lan T, Chen YC, Qian H (2016). Co-inhibition of pol theta and HR genes efficiently synergize with cisplatin to suppress cisplatin-resistant lung cancer cells survival. Oncotarget..

[53] McCabe N, Turner NC, Lord CJ, Kluzek K, Bialkowska A, Swift S (2006). Deficiency in the repair of DNA damage by homologous recombination and sensitivity to poly(ADP-ribose) polymerase inhibition. Cancer Res..

[54] Kim G, Ison G, McKee AE, Zhang H, Tang S, Gwise T (2015). FDA approval summary: olaparib monotherapy in patients with deleterious germline BRCA-mutated advanced ovarian cancer treated with three or more lines of chemotherapy. Clin Cancer Res..

[55] Pujade-Lauraine E, Ledermann JA, Selle F, Gebski V, Penson RT, Oza AM (2017). Olaparib tablets as maintenance therapy in patients with platinum-sensitive, relapsed ovarian cancer and a BRCA1/2 mutation (SOLO2/ENGOT-Ov21): a double-blind, randomised, placebo-controlled, phase 3 trial. Lancet Oncol..

[56] Lin ZP, Zhu YL, Lo YC, Moscarelli J, Xiong A, Korayem Y (2018). Combination of triapine, olaparib, and cediranib suppresses progression of BRCA-wild type and PARP inhibitor-resistant epithelial ovarian cancer. PLoS One..

[57] George E, Kim H, Krepler C, Wenz B, Makvandi M, Tanyi JL (2017). A patient-derived-xenograft platform to study BRCA-deficient ovarian cancers. JCI Insight..

[58] Morgan RD, Clamp AR, Evans DGR, Edmondson RJ, Jayson GC (2018). PARP inhibitors in platinum-sensitive high-grade serous ovarian cancer. Cancer Chemother Pharmacol..

[59] D'Andrea AD (2018). Mechanisms of PARP inhibitor sensitivity and resistance. DNA Repair (Amst)..

[60] Bitler BG, Watson ZL, Wheeler LJ, Behbakht K (2017). PARP inhibitors: clinical utility and possibilities of overcoming resistance. Gynecol Oncol..

[61] Sakai W, Swisher EM, Jacquemont C, Chandramohan KV, Couch FJ, Langdon SP (2009). Functional restoration of BRCA2 protein by secondary BRCA2 mutations in BRCA2-mutated ovarian carcinoma. Cancer Res..

[62] Makvandi M, Pantel A, Schwartz L, Schubert E, Xu K, Hsieh CJ (2018). A PET imaging agent for evaluating PARP-1 expression in ovarian cancer. J Clin Invest..

[63] Jiang X, Li X, Li W, Bai H, Zhang Z (2019). PARP inhibitors in ovarian cancer: sensitivity prediction and resistance mechanisms. J Cell Mol Med..

[64] Liu X, Han EK, Anderson M, Shi Y, Semizarov D, Wang G (2009). Acquired resistance to combination treatment with temozolomide and ABT-888 is mediated by both base excision repair and homologous recombination DNA repair pathways. Mol Cancer Res..

[65] Francica P, Rottenberg S (2018). Mechanisms of PARP inhibitor resistance in cancer and insights into the DNA damage response. Genome Med..

[66] Meghani K, Fuchs W, Detappe A, Drane P, Gogola E, Rottenberg S (2018). Multifaceted impact of microRNA 493-5p on genome-stabilizing pathways induces platinum and PARP inhibitor resistance in BRCA2-mutated carcinomas. Cell Rep..

[67] Moore K, Colombo N, Scambia G, Kim BG, Oaknin A, Friedlander M (2018). Maintenance olaparib in patients with newly diagnosed advanced ovarian cancer. N Engl J Med..

[68] Li S, Wang W, Niu T, Wang H, Li B, Shao L (2014). Nrf2 deficiency exaggerates doxorubicin-induced cardiotoxicity and cardiac dysfunction. Oxid Med Cell Longev..

[69] Korkmaz-Icoz S, Szczesny B, Marcatti M, Li S, Ruppert M, Lasitschka F (2018). Olaparib protects cardiomyocytes against oxidative stress and improves graft contractility during the early phase after heart transplantation in rats. Br J Pharmacol..

[70] Block WD, Yu Y, Lees-Miller SP (2004). Phosphatidyl inositol 3-kinase-like serine/threonine protein kinases (PIKKs) are required for DNA damage-induced phosphorylation of the 32 kDa subunit of replication protein A at threonine 21. Nucleic Acids Res..

[71] Zou L (2007). Single- and double-stranded DNA: building a trigger of ATR-mediated DNA damage response. Genes Dev..

[72] Cimprich KA, Cortez D (2008). ATR: an essential regulator of genome integrity. Nat Rev Mol Cell Biol..

[73] Adams BR, Golding SE, Rao RR, Valerie K (2010). Dynamic dependence on ATR and ATM for double-strand break repair in human embryonic stem cells and neural descendants. PLoS One..

[74] Matsuoka S, Rotman G, Ogawa A, Shiloh Y, Tamai K, Elledge SJ (2000). Ataxia telangiectasia-mutated phosphorylates Chk2 in vivo and in vitro. Proc Natl Acad Sci U S A..

[75] Bianco JN, Bergoglio V, Lin YL, Pillaire MJ, Schmitz AL, Gilhodes J (2019). Overexpression of Claspin and Timeless protects cancer cells from replication stress in a checkpoint-independent manner. Nat Commun..

[76] Zhang X, Lu X, Akhter S, Georgescu MM, Legerski RJ (2016). FANCI is a negative regulator of Akt activation. Cell Cycle..

[77] Pugliese GM, Salaris F, Palermo V, Marabitti V, Morina N, Rosa A, et al. Inducible SMARCAL1 knockdown in iPSC reveals a link between replication stress and altered expression of master differentiation genes. Dis Model Mech. 2019. 10.1242/dmm.039487 PMC6826020 31515241

[78] Yeom G, Kim J, Park CJ (2019). Investigation of the core binding regions of human Werner syndrome and Fanconi anemia group J helicases on replication protein A. Sci Rep..

[79] Zhou ZR, Yang ZZ, Wang SJ, Zhang L, Luo JR, Feng Y (2017). The Chk1 inhibitor MK-8776 increases the radiosensitivity of human triple-negative breast cancer by inhibiting autophagy. Acta Pharmacol Sin..

[80] Suzuki M, Yamamori T, Bo T, Sakai Y, Inanami O (2017). MK-8776, a novel Chk1 inhibitor, exhibits an improved radiosensitizing effect compared to UCN-01 by exacerbating radiation-induced aberrant mitosis. Transl Oncol..

[81] Kim HJ, Min A, Im SA, Jang H, Lee KH, Lau A (2017). Anti-tumor activity of the ATR inhibitor AZD6738 in HER2 positive breast cancer cells. Int J Cancer..

[82] Gamper AM, Rofougaran R, Watkins SC, Greenberger JS, Beumer JH, Bakkenist CJ (2013). ATR kinase activation in G1 phase facilitates the repair of ionizing radiation-induced DNA damage. Nucleic Acids Res..

[83] Reinhardt HC, Yaffe MB (2009). Kinases that control the cell cycle in response to DNA damage: Chk1, Chk2, and MK2. Curr Opin Cell Biol..

[84] Min A, Im SA, Jang H, Kim S, Lee M, Kim DK (2017). AZD6738, a novel oral inhibitor of ATR, induces synthetic lethality with ATM deficiency in gastric cancer cells. Mol Cancer Ther..

[85] Pabla N, Huang S, Mi QS, Daniel R, Dong Z (2008). ATR-Chk2 signaling in p53 activation and DNA damage response during cisplatin-induced apoptosis. J Biol Chem..

[86] Reaper PM, Griffiths MR, Long JM, Charrier JD, Maccormick S, Charlton PA (2011). Selective killing of ATM- or p53-deficient cancer cells through inhibition of ATR. Nat Chem Biol..

[87] Peasland A, Wang LZ, Rowling E, Kyle S, Chen T, Hopkins A (2011). Identification and evaluation of a potent novel ATR inhibitor, NU6027, in breast and ovarian cancer cell lines. Br J Cancer..

[88] Toledo LI, Murga M, Zur R, Soria R, Rodriguez A, Martinez S (2011). A cell-based screen identifies ATR inhibitors with synthetic lethal properties for cancer-associated mutations. Nat Struct Mol Biol..

[89] Mohni KN, Kavanaugh GM, Cortez D (2014). ATR pathway inhibition is synthetically lethal in cancer cells with ERCC1 deficiency. Cancer Res..

[90] Weber AM, Ryan AJ (2015). ATM and ATR as therapeutic targets in cancer. Pharmacol Ther..

[91] Foote KM, Lau A, Nissink JW (2015). Drugging ATR: progress in the development of specific inhibitors for the treatment of cancer. Future Med Chem..

[92] Knegtel R, Charrier JD, Durrant S, Davis C, O'Donnell M, Storck P (2019). Rational design of 5-(4-(isopropylsulfonyl)phenyl)-3-(3-(4-((methylamino)methyl)phenyl)isoxazol-5-yl )pyrazin-2-amine (VX-970, M6620): optimization of intra- and intermolecular polar interactions of a new ataxia telangiectasia mutated and Rad3-Related (ATR) kinase inhibitor. J Med Chem..

[93] Engelke CG, Parsels LA, Qian Y, Zhang Q, Karnak D, Robertson JR (2013). Sensitization of pancreatic cancer to chemoradiation by the Chk1 inhibitor MK8776. Clin Cancer Res..

[94] Huntoon CJ, Flatten KS, Wahner Hendrickson AE, Huehls AM, Sutor SL, Kaufmann SH (2013). ATR inhibition broadly sensitizes ovarian cancer cells to chemotherapy independent of BRCA status. Cancer Res..

[95] Kurmasheva RT, Kurmashev D, Reynolds CP, Kang M, Wu J, Houghton PJ, et al. Initial testing (stage 1) of M6620 (formerly VX-970), a novel ATR inhibitor, alone and combined with cisplatin and melphalan, by the Pediatric Preclinical Testing Program. Pediatr Blood Cancer. 2018;65(2). 10.1002/pbc.26825 PMC5876726 28921800

[96] Wengner AM, Siemeister G, Lucking U, Lefranc J, Wortmann L, Lienau P (2020). The novel ATR inhibitor BAY 1895344 is efficacious as monotherapy and combined with DNA damage-inducing or repair-compromising therapies in preclinical cancer models. Mol Cancer Ther..

[97] Buisson R, Boisvert JL, Benes CH, Zou L (2015). distinct but concerted roles of ATR, DNA-PK, and Chk1 in countering replication stress during S phase. Mol Cell..

[98] Jiang K, Pereira E, Maxfield M, Russell B, Goudelock DM, Sanchez Y (2003). Regulation of Chk1 includes chromatin association and 14-3-3 binding following phosphorylation on Ser-345. J Biol Chem..

[99] Niida H, Katsuno Y, Banerjee B, Hande MP, Nakanishi M (2007). Specific role of Chk1 phosphorylations in cell survival and checkpoint activation. Mol Cell Biol..

[100] Guzi TJ, Paruch K, Dwyer MP, Labroli M, Shanahan F, Davis N (2011). Targeting the replication checkpoint using SCH 900776, a potent and functionally selective CHK1 inhibitor identified via high content screening. Mol Cancer Ther..

[101] Ronco C, Martin AR, Demange L, Benhida R (2017). ATM, ATR, CHK1, CHK2 and WEE1 inhibitors in cancer and cancer stem cells. Medchemcomm..

[102] Paculova H, Kramara J, Simeckova S, Fedr R, Soucek K, Hylse O (2017). BRCA1 or CDK12 loss sensitizes cells to CHK1 inhibitors. Tumour Biol..

[103] Bahassi EM, Ovesen JL, Riesenberg AL, Bernstein WZ, Hasty PE, Stambrook PJ (2008). The checkpoint kinases Chk1 and Chk2 regulate the functional associations between hBRCA2 and Rad51 in response to DNA damage. Oncogene..

[104] Yang XH, Shiotani B, Classon M, Zou L (2008). Chk1 and Claspin potentiate PCNA ubiquitination. Genes Dev..

[105] Blasina A, Hallin J, Chen E, Arango ME, Kraynov E, Register J (2008). Breaching the DNA damage checkpoint via PF-00477736, a novel small-molecule inhibitor of checkpoint kinase 1. Mol Cancer Ther..

[106] King C, Diaz H, Barnard D, Barda D, Clawson D, Blosser W (2014). Characterization and preclinical development of LY2603618: a selective and potent Chk1 inhibitor. Invest New Drugs..

[107] Massey AJ, Stokes S, Browne H, Foloppe N, Fiumana A, Scrace S (2015). Identification of novel, in vivo active Chk1 inhibitors utilizing structure guided drug design. Oncotarget..

[108] Angius G, Tomao S, Stati V, Vici P, Bianco V, Tomao F. Prexasertib, a checkpoint kinase inhibitor: from preclinical data to clinical development. Cancer Chemother Pharmacol. 2019. 10.1007/s00280-019-03950-y 31512029

[109] Syljuasen RG, Sorensen CS, Hansen LT, Fugger K, Lundin C, Johansson F (2005). Inhibition of human Chk1 causes increased initiation of DNA replication, phosphorylation of ATR targets, and DNA breakage. Mol Cell Biol..

[110] Kawahara N, Ogawa K, Nagayasu M, Kimura M, Sasaki Y, Kobayashi H (2017). Candidate synthetic lethality partners to PARP inhibitors in the treatment of ovarian clear cell cancer. Biomed Rep..

[111] Ledford H (2016). US cancer institute to overhaul tumour cell lines. Nature..

[112] Kim H, George E, Ragland R, Rafail S, Zhang R, Krepler C (2017). Targeting the ATR/CHK1 axis with PARP inhibition results in tumor regression in BRCA-mutant ovarian cancer models. Clin Cancer Res..

[113] Sun C, Cao W, Qiu C, Li C, Dongol S, Zhang Z (2020). MiR-509-3 augments the synthetic lethality of PARPi by regulating HR repair in PDX model of HGSOC. J Hematol Oncol..

[114] Kondrashova O, Topp M, Nesic K, Lieschke E, Ho GY, Harrell MI (2018). Methylation of all BRCA1 copies predicts response to the PARP inhibitor rucaparib in ovarian carcinoma. Nat Commun..

[115] Gitto SB, Kim H, Rafail S, Omran DK, Medvedev S, Kinose Y (2020). An autologous humanized patient-derived-xenograft platform to evaluate immunotherapy in ovarian cancer. Gynecol Oncol..

[116] Liu HD, Xia BR, Jin MZ, Lou G. Organoid of ovarian cancer: genomic analysis and drug screening. Clin Transl Oncol. 2020. 10.1007/s12094-019-02276-8 PMC7316695 31939100

[117] Kopper O, de Witte CJ, Lohmussaar K, Valle-Inclan JE, Hami N, Kester L (2019). An organoid platform for ovarian cancer captures intra- and interpatient heterogeneity. Nat Med..

[118] Hill SJ, Decker B, Roberts EA, Horowitz NS, Muto MG, Worley MJ (2018). Prediction of DNA repair inhibitor response in short-term patient-derived ovarian cancer organoids. Cancer Discov..

[119] Saldivar JC, Cortez D, Cimprich KA (2017). The essential kinase ATR: ensuring faithful duplication of a challenging genome. Nat Rev Mol Cell Biol..

[120] Yazinski SA, Comaills V, Buisson R, Genois MM, Nguyen HD, Ho CK (2017). ATR inhibition disrupts rewired homologous recombination and fork protection pathways in PARP inhibitor-resistant BRCA-deficient cancer cells. Genes Dev..

[121] Fang P, De Souza C, Minn K, Chien J (2019). Genome-scale CRISPR knockout screen identifies TIGAR as a modifier of PARP inhibitor sensitivity. Commun Biol..

